# Interaction of Health Literacy and Problematic Mobile Phone Use and Their Impact on Non-Suicidal Self-Injury among Chinese Adolescents

**DOI:** 10.3390/ijerph16132366

**Published:** 2019-07-03

**Authors:** Danlin Li, Rong Yang, Yuhui Wan, Fangbiao Tao, Jun Fang, Shichen Zhang

**Affiliations:** 1 Department of Maternal and Child Health, School of Public Health, Anhui Medical University, 81th Meishan Road, Hefei 230032, Anhui Province, China; 2 Anhui Provincial Key Laboratory of Population Health & Aristogenics, 81th Meishan Road, Hefei 230032, Anhui Province, China; 3 Department of Toxicology, School of Public Health, Anhui Medical University, 81th Meishan Road, Hefei 230032, Anhui Province, China; 4 Faculty of Pharmaceutical Science, Sojo University, Ikeda 4-22-1, Kumamoto 860-0082, Japan

**Keywords:** interaction, health literacy, problematic mobile phone use, non-suicidal self-injury, adolescents

## Abstract

Non-suicidal self-injury (NSSI) is prevalent among adolescents. Low health literacy (HL) and problematic mobile phone use (PMPU) are risk factors of NSSI. But so far, no study has examined the interactive role of HL and PMPU on NSSI. In this context, the present study aimed to examine the interactions of HL and PMPU and their impact on NSSI in a school-based sample of Chinese adolescents. A total of 22,628 junior and high school students (10,990 males and 11,638 females) were enrolled in this study. The outcomes were self-reported HL, PMPU and NSSI. Analyses were conducted with chi-square tests and logistic regression models. The prevalence of NSSI was 32.1%. Low HL and PMPU were significantly associated with NSSI independently (OR_low HL_ = 1.886, 95% CI = 1.723–2.065, OR_PMPU_ = 2.062, 95% CI = 1.934–2.199). Interaction analysis indicated that low HL and PMPU were interactively associated with increased risks of NSSI (OR = 2.617, 95% CI = 2.375–2.884). In all, our findings indicate that HL and PMPU are associated with NSSI independently and interactively. The intervention programs of NSSI should consider the adolescents HL levels and PMPU.

## 1. Introduction

Non-suicidal self-injury (NSSI) refers to the intentional self-inflicted destruction of body tissue without suicidal intention and for purposes not socially sanctioned. The forms of NSSI include cutting, skin carving, burning, severe scratching/abrading, and punching/hitting [1]. Existing evidences found that NSSI increases the risk of suicide leading to death, and NSSI usually first emerges during early adolescence [2,3,4]. Moreover, adolescents appear to engage in NSSI at higher rate than adults [5]. A study in Spanish suggested approximately more than half of adolescents reported a history of NSSI at least once in the past 12 months, and 32.2% had serve NSSI behaviors [6]. Swannell et al. estimated that the international rate of NSSI among adolescents was 17.2% [7]. Furthermore, a meta-analysis based on Chinese adolescents reported that the pooled prevalence of NSSI was 22.37% [8]. In general, NSSI is still highly prevalent among adolescents and is a serious public health problem [9,10]. 

Health literacy (HL) is defined as “how well a person can get and understand the health information and services, and use them to make good health decisions” [11]. Furthermore, the field of HL includes three types that functional literacy, interactive literacy and critical literacy. In 2008, Nutbeam proposed that HL is a more advanced cognitive and literacy skill which can be used to participate activities actively in everyday and apply new information to the changing circumstances. This definition emphasized that HL is made up of a set of skills [12]. In recent years, HL has become an important topic in promoting health. All over the world, the rate of adequate HL in different countries is diverse. A HL survey conducted in Mashhad noted that about 11.5% of adolescents had adequate HL [13]. In 2017, China Health Education Center reported that 15.58% of adolescents aged 15–24 years had adequate HL. Inadequate HL in adolescents was associated with poor health outcomes such as low medication adherence, psychological symptoms, poor physical health and so on [14,15,16]. Also, poor HL was reported to be associated with NSSI, obesity, alcohol abuse and others health risky behaviors among adolescents [14,17,18].

With the progress of internet, mobile phone becomes an indispensable tool in people’s life. However, how to use mobile phone properly is becoming critically important that cannot be ignored, especially in adolescents. Studies from multiple countries, such as China, Korea, and India, showed that the rate of mobile phone addiction in adolescents was 14%–29.2% [19,20,21]. Many researchers reported that problematic mobile phone use (PMPU) could cause poor sleep quality, unintentional injuries, depression, alcohol use, and suicidal tendencies [22,23,24,25]. However, most of those studies only focused on the independent effects of PMPU on physiology and psychology, but did not investigated the interactive role of HL and PMPU.

In psychological events, many interdependent events constitute an inter-behavioral fields which can be influenced by many factors, each of which is interrelated [26]. The occurrence of psychological events are the result of synergy between various factors in the interaction fields [26,27]. Not surprisingly, interaction does exist between HL and PMPU. Most of the adolescents with higher rate of using cell phone have limited HL score [28,29]. However, the interactions of HL and PMPU and their impact on NSSI are largely unknown. So, current study proposes two hypotheses and validates.

(1) Associations of HL and PMPU with NSSI respectively;

(2) Whether the interaction of HL and PMPU increased the possibility of NSSI among Chinese adolescents.

## 2. Methods

### 2.1. Study Participants and Procedures

The study was conducted in accordance with the Declaration of Helsinki, and the protocol was approved by the Ethics Committee of Anhui Medical University (1 March 2014; approval number 20140087). All subjects participated in the study upon receiving informed consent form their parents. In the whole investigation, if they were not willing to participate, they could withdraw from the study anytime. The participants were recruited from junior and senior high schools located in six cities in China, including both urban and rural regions, by using multistage stratified cluster sampling. Firstly, six cities were select by convenient sampling. These cities were Shenyang (capital of Liaoning Province), Xinxiang (North of Henan Province), Yangjiang (Southwest coast of Guangdong Province), Chongqing (one of China’s four direct-controlled municipalities), Ulanchap (Central Inner Mongolia Autonomous Region) and Bengbu (Northeastern of Anhui province). Then, eight schools (two rural junior and two senior schools, two urban junior and two senior schools) were selected in each region based on the stratified cluster sampling. Lastly, four to six classes were selected randomly from each grade in each school. Data for this study were collected from November 2015 to January 2016.

Under the supervision of teachers, each participant completed a self-report questionnaire, including socio-demographic variables, HL, PMPU and NSSI, during 20–30 min in the classroom. In total, 23,137 students took part in this survey. Excluding the incomplete questionnaires, there were 22,628 valid questionnaires with the efficiency rate of 97.8%. The mean age of the participants was 15.36 (SD = 1.79).

### 2.2. Demographic Information

In this study, socio-demographic variables recorded were as follows: Age, gender (male or female), grade (junior or senior high schools), any siblings (yes or no), registered residence (urban or rural), accommodation type (boarding student or commuting student), self-reported family economic situation (bad, general, or good), parents’ educational level (<high school degree or ≥high school degree) and number of friends (≤ 2, 3–5, or ≥ 6).

### 2.3. Questionnaire Data

The measurement of HL was based on the Chinese Adolescent Interactive Health Literacy Questionnaire (CAIHLQ). This scale assesses six dimensions (physical activities, interpersonal relationship, stress management, self-actualization, health awareness, dietary behavior) of HL with 31 items (e.g., “follow a planned exercise program”, “take time with your family or friends”, “balance time between study and play”, “feel each day is very meaningful”, “containing sugars and food continuing sugar”, “eat 200–400g of fresh fruit each day”). To each question, participants selected an answer from five answer categories (never and no desire, never but with desire, occasionally and irregularly, often, and routinely). Previous study has proved that the questionnaire has good reliability and validity [30]. In this study, an internal consistency test showed that the Cronbach’s α was 0.910, Cronbach’s α of each dimension was 0.662 to 0.847. For each participant’s total score, the scores ranged between 31 and 155. We identified three categories of the score: Low (< *P*_25_), middle (*P*_25_–*P*_75_) and high (> *P*_75_), respectively.

PMPU was measured by the Self-rating Questionnaire for Adolescent Problematic Mobile Phone Use (SQAPMPU) [31]. It comprised 13 items that responded to a 5-point Likert scale (never, occasionally, sometimes, often, and always) and covered three dimensions including six questions for withdrawal symptoms (e.g., “If I don’t have a phone, I will feel overwhelmed”), four questions for craving (e.g., “I always feel that I don’t have enough time to use my phone”), three questions for physical and mental health status (e.g., ‘Too much mobile phone use leads to insufficient sleep’). Exploratory factor analysis showed the variance cumulative contribution rate of this questionnaire was 59.13%, and Cronbach’s alpha coefficient was 0.87 [31]. In this study, the Cronbach’s alpha coefficient was 0.923. The total score ranged from five to 65, with higher scores indicating PMPU (≥ P_75_). Therefore, the students with scores ≥ 28 were defined as problematic mobile phone users. 

NSSI of the participants over the previous 12 months before the survey were assessed by the self-report questionnaire, which included eight items [32]. All the response options were “yes” or “no”. The details of the questions were as follows: (1) Have you ever hit yourself?; (2) have you ever pulled your hair yourself?; (3) have you ever banged your head or fist against something?; (4) have you ever pinched or scratched yourself?; (5) have you ever bitten yourself?; (6) have you ever cut or pierced yourself?; (7) have you ever exposed yourself to smoke, fire, and flames or come in contact with heat and hot substances?; and (8) have you ever ingested a toxic substance or object? As long as the answer was “yes” (one or more times), the student will be judged as having NSSI behaviors. The Cronbach’s α coefficient for NSSI in the present was 0.779, which was similar to previous research [33].

### 2.4. Statistical Analysis

In this study, all analyses were conducted with SPSS software version 23.0 (SPSS Inc, Chicago, IL, USA). The chi-square test was applied to compare the incidence of NSSI among different demographic variables. Multivariate logistic regression models were used to examine the associations of NSSI, HL and PMPU and to evaluate the interaction of HL and PMPU with NSSI. Adjustment was made for confounding factors such as gender, grade, registered residence, accommodation type, parents’ educational level, self-reported family economical, number of friends and city. Odds ratios (OR) and 95% confidence intervals (CI) for the factors were calculated to present the associations. In the analyses, *p* < 0.05 was considered statistically significant.

## 3. Results

### 3.1. Univariate Analyses

Table 1 presented the frequency characteristics and group differences of the sample in current study. Of the 22,628 students, 10,990 were males (48.6%) and 11,638 were females (51.4%), and the mean age was 15.36 years (SD = 1.79). The overall CAIHLQ mean score for all participants was 104.06 ± 18.68, and the value of *P*_25_ and *P*_75_ were 92, and 116, respectively. Overall, 7261 (32.1%) students reported NSSI behaviors in the past 12 months. The males had higher incidence rate of NSSI than the females (male (35.2%) vs. female (29.1%), *p* < 0.001). Furthermore, statistical significance was found on grade, registered residence, accommodation type, parents’ educational level, self-reported family economy, number of friends and city (*p* < 0.05 for each), while NSSI revealed no statistically significant differences by any siblings (*p* > 0.05, Table 1). In addition, NSSI was more likely to occur in students with lower HL than those with higher HL (high HL (22.9%) vs. medium HL (33.1%) vs. low HL (39.2%), *p* < 0.001). The occurrence of NSSI was also significantly higher in students with PMPU (no (27.6%) vs. yes (45.4%), *p* < 0.001, Table 1).

The prevalence of all kinds of NSSI by gender is shown in Table 2. The rate of four types of NSSI was more than 10%, i.e., banging head (4510 (19.9%)), hitting (3203 (14.2%)), scratching (2710 (12.0%)) and pulling hair (2622 (11.6%)). Moreover, the differences between males and females for each behavior were statistically significant. Compared with the males, the females were more likely to engage in scratching (1650 (7.3%); *χ*^2^ = 110.153), banging head (1634 (7.2%); *χ*^2^ = 48.302) and hitting (1516 (6.7%); *χ^2^* = 25.124), but males were more likely to engage in banging head (2827 (12.7%); *χ*^2^ = 521.060), hitting (1687 (7.5%); *χ*^2^ = 25.124), pulling hair (1558 (5.1%); *χ*^2^ = 139.819) (*p* < 0.01 for each, Table 2).

### 3.2. Multivariate Logistic Regression Analyses

Results from multivariate logistic regression analysis indicated that both HL (OR_medium_ = 1.609, 95% CI: 1.489–1.738, OR_low_ = 1.886, 95% CI: 1.723–2.065,) and PMPU (OR = 2.062, 95% CI: 1.934–2.199) remained independently associated with NSSI (*p* < 0.001 for each, Table 3). Besides, they had a multiplied interaction impact on NSSI. After adjusting for gender, grade, registered residence, accommodation type, parents’ educational level, self-reported family economic situation, number of friends and city, these positive associations remained significant (Table 3).

**Figure 1:**
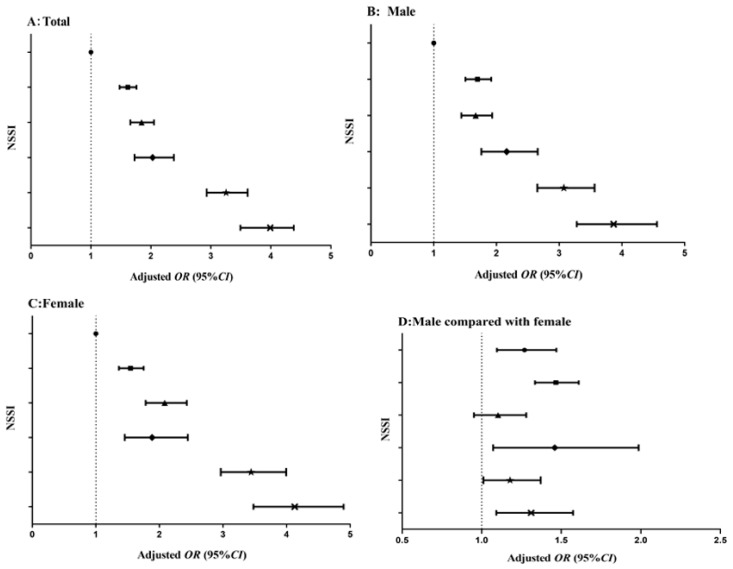
Odds ratio (95% CI) associated with the interaction of HL and PMPU on NSSI in male and female, and the gender ratio. OR is odds ratio; CI is confidence interval; HL is health literacy; NSSI is non-suicidal self- injury; PMPU is problematic mobile phone use; adjusted for gender, grade, registered residence, accommodation type, parents’ educational level, self-reported family economic situation, number of friends and city. (**A**) is total; (**B**) is male; (**C**) is female; (**D**) is gender comparison (male compared with female). ● No PMPU + High HL (the students with high HL and no PMPU); ■ no PMPU + medium HL; ▲ no PMPU + low HL; ◆ have PMPU + high HL; ★ have PMPU + medium HL; **×** have PMPU + low HL.

Figure 1 showed the results of the different groups of HL and PMPU in all students, males, females and the gender comparison. In all students, the multiple logistic regression model indicated students with both low HL and PMPU had the highest risks of NSSI (OR = 3.253, 95% CI: 2.930–3.613) (Figure 1A). Similar results were found in males and females (OR_males_ = 3.074, 95% CI: 2.652–3.564, OR_females_ = 3.441, 95% CI: 2.965−3.993) (Figure 1B,C). Besides, in the gender comparison, the results suggested that males had a higher risk of NSSI than females, except for the group of students with low HL and no PMPU (Figure 1D). For more details check Appendix A Table A1.

## 4. Discussion

NSSI is a serious health hazard to adolescents mental health. Because of the definition of NSSI, samples and assessment tools are different, the self-reported NSSI ranged from 7.9% to 73.7% among adolescents in the word [34,35,36,37]. In current study, the rate of NSSI was 32.1%, which is higher than previous studies in China over the past five years (24.9%–29.0%) [38,39]. Obviously, the rate of NSSI among adolescents is increasing, it is thus of great importance to take the necessary measures to prevent and control the incidence of NSSI. Furthermore, we found that middle school students reported a higher rate of NSSI than high school, that means, NSSI was more likely to happen in early adolescence, which is similar to other studies [4,32]. Poon et al. proposed that students may show higher reward system sensitivities in early adolescence, making them more likely to participate in NSSI [40]. Furthermore, in late adolescence, NSSI sometimes can be taken over by other dysfunctional behaviors such as substance abuse [41]. The different rate of NSSI between urban and rural students might be due to the economic development status, parental care-giving, or other social factors. In addition, compared with those who have less friends, the students who have more friends showed a lower incidence rate of NSSI. These results suggested that peer relationship and interpersonal instability play an important function in adolescence development [42]. All in all, a series of results of demographic characteristics were consistent with social ecological theory. Families, schools, peers, etc., all had an impact on adolescents [43,44].

Regarding gender differences, we found that NSSI was more likely to occur in males, which was consistent with some previous studies [32,45], but there were also studies showing the opposite results [34,46]. This may relate to the impulsive personality that is significantly associated with males [47]. Some previous studies found that self-cutting or hitting was the most common way of NSSI [39,48]. However, our findings indicated that banging a head or fisting against something was the most frequently reported method of NSSI in Chinese adolescents. Cultural and evaluation criteria differences may be accounting for this difference. Females tended to pinch or scratch themselves in more cases, whereas males seemed to bang their heads more than females. The mechanism for causing this difference remains to be explored in the future.

Bandura noted that cognitive factors play an important role in the decision-making process [49]. The concept is consistent with the dual-process models, indicating that the person with lower HL levels has a lower ability to make rational decisions. Therefore, they are more likely to make reactive decisions and take risk behaviors [50]. What’s more, the existing evidence showed that low HL was related to NSSI [14], this relationship has also been verified in this research. Our results indicated that NSSI was more likely to occur in students with low HL. The possible reason was that adolescents may be ill-equipped to recognize signs of negative emotion and to respond due to low HL, so they take to NSSI to eliminate negative emotions [51].

Published studies have found that smoking, alcohol consumption, drug addiction and other addictive behaviors are associated with NSSI [52,53]. However, in our research, we chose the PMPU which is more common with rapid development of technology. Additionally, some studies reported that frequent mobile phone use was associated with suicidal feelings and self-injury, this in line with our results [54,55]. Oshima et al. has pointed out that the reason why using a mobile phone could increase the risk of self-injury was that it could bring negative emotions or stress [55]. Moreover, in current study, low HL and PMPU increased risk of NSSI both independently and interactively (Figure 1 and Table 3). The multivariate logistic regression analyses indicated that students with low HL were likely to experience NSSI, and the association was enhanced by PMPU. This result was consistent with the inter-behavioral fields of developmental psychology [26,27]. These findings indicated that the role of HL and PMPU should be noted in Chinese adolescents. Understanding these interactions will contribute to enhancing and promoting advances in development of prevention strategies for NSSI. However, further investigations are needed.

This study was a representative nationwide epidemiologic study with large samples. However, there are several limitations to this study. Firstly, we used a cross-sectional design, so causal relationships were not defined. Secondly, we used self-reported data, therefore, recall and reporting bias might be inevitable. Finally, missing school students were not included in the survey, and these students may have more behavioral problems. Further studies should consider a prospective cohort design to clarity causal relationship of HL and PMPU with NSSI and as much as possible to include the adolescents outside the school.

## 5. Conclusions

The findings of our study indicate that HL and PMPU are associated with NSSI, both independently and interactively. HL and PMPU should thus be considered in intervention programs for the aim of reducing the rate of NSSI among adolescents.

## Figures and Tables

**Table 1:** Frequency characteristics of non-suicidal self-injury (NSSI) in Chinese adolescents (%).

Variable	Total Sample (*n* = 22,628)	NSSI		*χ* ^2^	φ/V
		No (*n* = 15,367)	Yes (*n* = 7261)		
Gender				96.332 ***	−0.065 ***
Male	10990 (48.6)	7119 (64.8)	3871 (35.2)		
Female	11638 (51.4)	8248 (70.9)	3390 (29.1)		
Grade				65.487 ***	−0.054 ***
Middle school	11993 (53.0)	7861 (65.6)	4132 (34.4)		
High school	10635 (47.0)	7506 (70.6)	3129 (29.4)		
Registered residence				26.357 ***	−0.034 ***
Rural	10882 (48.1)	7210 (66.3)	3672 (33.7)		
Urban	11746 (51.9)	8157 (69.4)	3589 (30.6)		
Any siblings				0.239	0.003
Yes	12908 (57.0)	8749 (67.8)	4159 (32.2)		
No	9720 (43.0)	6618 (68.1)	3102 (31.9)		
Accommodation type				6.952 **	−0.018 **
Boarding student	11320 (50.0)	7595 (67.1)	3725 (32.9)		
Commuting student	11308 (50.0)	7772 (68.7)	3536 (31.3)		
Father’s educational level ^a^				8.823 **	−0.020 **
< High school degree	13006 (57.5)	8735 (67.2)	4271 (32.8)		
≥ High school degree	9424 (41.6)	6506 (69.0)	2918 (31.0)		
Mother’s educational level ^b^				9.751 **	−0.021 **
< High school degree	14335 (63.4)	9639 (67.2)	4696 (32.8)		
≥ High school degree	8105 (35.8)	5614 (69.3)	2491 (30.7)		
Self-reported family economy				67.759 ***	0.055 ***
Bad	3240 (14.3)	2005 (61.9)	1235 (38.1)		
General	16345 (72.2)	11316 (69.2)	5029 (30.8)		
Good	3043 (13.4)	2046 (67.2)	997 (32.8)		
Number of friends				37.753 ***	0.041 ***
≤ 2	5514 (24.4)	3560 (64.6)	1954 (35.4)		
3-5	9620 (42.5)	6622 (68.8)	2998 (31.2)		
≥ 6	7494 (33.1)	5185 (69.2)	2309 (30.8)		
City				102.702 ***	0.067 ***
Shenyang	3217(14.2)	2165(67.3)	1052(32.7)		
Xinxiang	3230(14.3)	2327(72.0)	903(28.0)		
Yangjiang	5061(22.4)	3525(69.7)	1536(30.3)		
Chongqing	5588(24.7)	3865(69.2)	1723(30.8)		
Ulanchap	2333(10.3)	1506(64.6)	827(35.4)		
Bengbu	3199(14.1)	1979(61.9)	1220(38.1)		
HL				343.427 ***	0.123 ***
High	5486 (24.2)	4230 (77.1)	1256 (22.9)		
Medium	11842 (52.3)	7917 (66.9)	3925 (33.1)		
Low	5300 (23.4)	3220 (60.8)	2080 (39.2)		
PMPU				623.184 ***	0.166 ***
No	16876 (74.6)	12224 (72.4)	4652 (27.6)		
Yes	5752 (25.4)	3143 (54.6)	2609 (45.4)		

Statistical methods: Chi-square test, φ/V is the effect size; HL is health literacy; NSSI is non-suicidal self- injury; PMPU is problematic mobile phone use; *** *p* < 0.001, ** *p* < 0.01; ^a^ 198 students have no father; ^b^ 188 students have no mother.

**Table 2:** Proportion of different NSSI behaviors in adolescents by gender.

Items	Total Sample (*n* = 22628)	Male (*n* = 10990)	Female (*n* = 11638)	*χ* ^2^	*φ*/*V*
1.Have you ever hit yourself?				25.124 ***	−0.033 ***
No	19425 (85.8)	9303 (41.1)	10122 (44.7)		
Yes	3203 (14.2)	1687 (7.5)	1516 (6.7)		
2. Have you ever pulled your hair yourself?				139.819 ***	−0.079 ***
No	20006 (88.4)	9432 (41.7)	10574 (46.7)		
Yes	2622 (11.6)	1558 (6.9)	1064 (4.7)		
3. Have you ever banged your head or fist against something?				521.060 ***	−0.152 ***
No	18118 (80.1)	8114 (35.9)	10004 (44.2)		
Yes	4510 (19.9)	2876 (12.7)	1634 (7.2)		
4. Have you ever pinched or scratched yourself?				110.153 ***	0.070 ***
No	19918 (88.0)	9930 (43.9)	9988 (44.1)		
Yes	2710 (12.0)	1060 (4.7)	1650 (7.3)		
5. Have you ever bitten yourself?				48.302 ***	0.046 ***
No	21044 (93.0)	10354 (45.8)	10690 (47.2)		
Yes	1584 (7.0)	636 (2.8)	948 (4.2)		
6. Have you ever cut or pierced yourself?				32.246 ***	0.038 ***
No	21277 (94.0)	10435 (46.1)	10842 (47.9)		
Yes	1351 (6.0)	555 (2.5)	796 (3.5)		
7. Have you ever exposed yourself to smoke, fire and flames or come in contact with heat and hot substances?				51.465 ***	−0.048 ***
No	21692 (95.9)	10428 (46.1)	11264 (49.8)		
Yes	936 (4.1)	562 (2.5)	374 (1.7)		
8. Have you ever ingested a toxic substance or object?				48.089 ***	−0.046 ***
No	22332 (98.7)	10787 (46.7)	11545 (51.0)		
Yes	296 (1.3)	203 (0.9)	93 (0.4)		

Statistical methods: Chi-square test; φ/V is effect size; *** *P* < 0.001.

**Table 3:** Associations of HL, PMPU and NSSI in Chinese adolescents.

Variables	NSSI		
	*n (%)*	Crude OR (95 % CI)	Adjusted OR (95 % CI) ^a^
HL			
High	5415 (23.9)	1.000	1.000
Medium	11686 (51.6)	1.572 (1.459–1.693) ***	1.609 (1.489–1.738) ***
Low	5190 (22.9)	1.878 (1.724–2.045) ***	1.886 (1.723–2.065) ***
PMPU			
No	16644 (73.6)	1.000	1.000
Have	5647 (25.0)	2.027 (1.904–2.159) ***	2.062 (1.934–2.199) ***
HL × PMPU			
High × No		1.000	1.000
Medium × Have		2.108 (1.946–2.283) ***	2.170 (2.000–2.354) ***
Low × Have		2.632 (2.395–2.891) ***	2.617 (2.375–2.884) ***

OR is odds ratio; CI is confidence interval; HL is health literacy; NSSI is non-suicidal self- injury; PMPU is problematic mobile phone use; *** *p* < 0.001 compared with referent; ^a^ adjusted for gender, grade, registered residence, accommodation type, parents’ educational level, self-reported family economic situation, number of friends and city.

## Appendix A

**Table A1:** Odds ratio (95 % CI) associated with the interaction of HL and PMPU on NSSI in males and females, and the gender ratio.

Groups	*n* (%)	Crude OR (95 % CI) ^A^	Adjusted OR (95 % CI) ^a^	*n* (%)	Crude OR (95 % CI) ^B^	Adjusted OR (95 % CI) ^a^	*n* (%)	Crude OR (95 % CI) ^C^	Adjusted OR (95 % CI) ^a^	Crude OR (95 % CI^)^^D^	Adjusted OR (95 % CI) ^a^
No + High	4630 (20.5)	1.000	1.000	2255 (20.5)	1.000	1.000	2375 (20.4)	1.000	1.000	1.221 (1.059−1.408) **	1.269 (1.096−1.469) ***
No + Medium	8923 (39.4)	1.589 (1.461−1.729) ***	1.614 (1.479−1.760) ***	4120 (37.5)	1.761 (1.565−1.982) ***	1.698 (1.505−1.916) ***	4803 (41.3)	1.456 (1.290−1.643) ***	1.545 (1.363−1.752) ***	1.447 (1.348−1.619) ***	1.466 (1.335−1.610) ***
No + Low	3323 (15.7)	1.853 (1.674−2.051) ***	1.844 (1.657−2.051) ***	1699 (15.5)	1.782 (1.547−2.052) ***	1.668 (1.440−1.933) ***	1624 (14.0)	1.920 (1.658−2.224) ***	2.082 (1.784−2.429) ***	1.133 (0.980−1.311)	1.103 (0.951−1.280)
Yes + High	856 (3.8)	2.060 (1.760−2.412) ***	2.028 (1.727−2.381) ***	512 (4.7)	2.209 (1.802−2.707) ***	2.162 (1.760−2.657) ***	344 (3.0)	1.739 (1.349−2.242) ***	1.885 (1.454−2.445) ***	1.551 (1.158−2.079) **	1.459 (1.072−1.986) *
Yes + Medium	2919 (12.9)	3.133 (2.830−3.470) ***	3.253 (2.930−3.613) ***	1383 (12.6)	3.162 (2.736−3.655) ***	3.074 (2.652−3.564) ***	1536 (13.2)	3.138 (2.717−3.623) ***	3.441 (2.965−3.993) ***	1.231 (1.063−1.425) **	1.178 (1.011−1.371) *

OR is odds ratio; CI is confidence interval; *** *p* < 0.001 compared with referent; HL is health literacy; PMPU is problematic mobile phone use; A is total; B is male; C is female; D is gender comparison (male compared with female); ^a^ adjusted for gender, grade, registered residence, accommodation type, parents’ educational level, self-reported family economic situation, number of friends and city.
